# A Compass to Guide Insights into T_H_17 Cellular Metabolism and Autoimmunity

**DOI:** 10.20900/immunometab20220001

**Published:** 2021-11-18

**Authors:** Adrianna N. Wilson, Sarah A. Mosure, Laura A. Solt

**Affiliations:** 1 Department of Immunology and Microbiology, The Scripps Research Institute, Jupiter, FL 33458, USA; 2 Skaggs Graduate School of Chemical and Biological Sciences, The Scripps Research Institute, Jupiter, FL 33458, USA; 3 Department of Integrative Structural and Computational Biology, The Scripps Research Institute, Jupiter, FL 33458, USA; 4 Department of Molecular Medicine, The Scripps Research Institute, Jupiter, FL 33458, USA

**Keywords:** T_H_17 cell, T regulatory, Foxp3, metabolism, glycolysis, inflammation, arginine, polyamine

## Abstract

T cells rapidly convert their cellular metabolic requirements upon activation, switching to a highly glycolytic program to satisfy their increasingly complex energy needs. Fundamental metabolic differences have been established for the development of Foxp3^+^ T regulatory (Treg) cells versus T_H_17 cells, alterations of which can drive disease. T_H_17 cell dysregulation is a driver of autoimmunity and chronic inflammation, contributing to pathogenesis in diseases such as multiple sclerosis. A recent paper published in *Cell* by Wagner, et al. combined scRNA-seq and metabolic mapping data to interrogate potential metabolic modulators of T_H_17 cell pathogenicity. This Compass to T_H_17 cell metabolism highlights the polyamine pathway as a critical regulator of T_H_17/Treg cell function, signifying its potential as a therapeutic target.

T helper 17 (T_H_17) cells differentiate from naïve CD4^+^ T cells in the presence of TGFβ and pro-inflammatory cytokines, such as IL-6, IL-1β, or IL-21, to control infections from fungi and extracellular bacteria [1,2]. Foxp3^+^ T regulatory (Treg) cells also differentiate in the presence of TGFβ [1]. The balance between T_H_17 and Treg cells can separate a normal immune response from the development of autoimmunity and chronic inflammation, including rheumatoid arthritis (RA) and multiple sclerosis (MS) [2,3]. To add another layer of complexity, T_H_17 cells have varying degrees of pathogenicity, as determined by their transcriptional signatures and secreted cytokine profiles [4–6]. “Pathogenic” T_H_17 cells secrete higher levels of interferon gamma (IFN*γ*), granulocyte-macrophage colony-stimulating factor (GM-CSF), CXCL3, and IL-22, whereas “non-pathogenic” T_H_17 cells secrete more IL-10 and IL-21 [7,8]. The “pathogenic fate” of T_H_17 cells depends on the cytokine milieu present at the time of T cell activation; IL-6 and TGFβ steer T_H_17 cells toward a “non-pathogenic” fate, whereas the addition of IL-1β and IL-23 increase T_H_17 cell pathogenicity [4,6,8]. Thus, T_H_17 plasticity appears to be determined by environmental signals and cytokines present in the milieu [8,9].

Cellular metabolism has also been reported to underlie T_H_17 cell pathogenicity and affect the T_H_17/Treg balance. Pathogenic T_H_17 (T_H_17p) cells require more energy than naïve T cells, non-pathogenic T_H_17 (T_H_17n), and Treg cells, which rely on fatty acid oxidation (FAO) for cellular ATP [1,10]. T_H_17p cells utilize glycolysis and oxidative phosphorylation for their energetic needs, possibly because this process can generate energy faster than FAO [10]. Identification of transcriptional differences in immune cell populations have been facilitated by the advent of single cell sequencing technologies. However, techniques for high resolution single cell metabolomic profiling are underdeveloped. Metabolites undergo rapid alterations in composition and abundance, and many are present in trace amounts. Limitations in both detection and biological annotation leave much unknown about cellular metabolism [11]. The studies that have been performed, mainly on well characterized pathways and metabolites, underscores the importance for understanding cellular metabolism in the immune system and how infection and therapeutic administration can alter it [12,13].

To address the current limitations in metabolomics research, Wagner et al. developed Compass, a flux balance analysis (FBA) algorithm utilizing single cell transcriptomics to map cellular contexts and predict metabolic states [14]. In this algorithm, each metabolic reaction is assigned a score based on the cellular environment’s ability to maintain it. Catalytic enzyme mRNA levels, reaction stoichiometry, individual and neighboring cell states are considered when assigning a reaction’s “potential activity”. Therefore, Compass generates a “quantitative” metabolic profile for every cell that is analyzed. To test Compass, the authors used previously published scRNA-seq on T_H_17p and T_H_17n [9,15]. Using principle component analysis (PCA) of the calculated “activity” of meta metabolic-reactions, the authors found that overall metabolic activity and T effector functions were the main determinants of heterogeneity in the T_H_17 cell populations. Moreover, Compass successfully predicted that T_H_17p cells were more glycolytic than T_H_17n cells and that T_H_17n cells utilize FAO for their ATP source [16,17]. Interestingly, within central carbon metabolism pathways, individual reactions were predicted to be both pro-pathogenic and pro-regulatory, highlighting individual reaction analysis as a strength of Compass. Collectively, these data attest to the robustness of their Compass algorithm.

Compass revealed the polyamine metabolic pathway was significantly associated with differences in T_H_17 pathogenicity. Exploring this finding further, the authors used qPCR to examine differences in gene expression of enzymes critical for the polyamine pathway. Spermidine/spermine N1 acetyltransferase (SAT1) and Ornithine Decarboxylase 1 (ODC1) are two rate-limiting enzymes important for putrescine biosynthesis and recycling. SAT1 was upregulated in T_H_17p cells over T_H_17n and Treg cells. ODC1 was expressed similarly in T_H_17n and T_H_17p but was significantly lower in Treg cells. Polyamine metabolites were then quantified using liquid chromatography-mass spectrometry (LC/MS) metabolomics. T_H_17p cells had higher levels of putrescine than T_H_17n cells, while cellular metabolites directly upstream and downstream of putrescine were consistent across T_H_17 cell subtypes. Through targeted metabolomic labeling and tracing experiments, they found T_H_17p cells preferentially synthesize or recycle polyamines. Collectively, this data demonstrated that the polyamine pathway, and specifically the biosynthesis of putrescine, may be associated with the functional state of T_H_17 cells.

To further interrogate the potential role of differential polyamine metabolism in T_H_17 cells, polyamine inhibitors were applied in vitro to observe their effects on T_H_17 cell differentiation. Difluoromethylornithine (DFMO), an irreversible inhibitor of ODC1, suppressed polyamines and canonical T_H_17 cytokine expression. DMFO administration also decreased expression of the T_H_17 transcriptional modulators ROR*γ*t and phosphorylated STAT3 in T_H_17p cells but not T_H_17n cells; however, it was interesting to note that the level of RORγt in control T_H_17n cells was low. Reciprocally, DMFO treatment increased Foxp3, the lineage defining transcription factor (TF) for Treg cells, in T_H_17n cells. These effects were recapitulated during differentiation of *Odc1*^−/−^ T cells and could be rescued by the administration of putrescine. Bulk RNA-seq experiments of T_H_17p, T_H_17n, and Treg cells confirmed that DMFO drove more of a Treg-specific transcriptome in T_H_17p and T_H_17n cells. Measurement of chromatin accessibility by ATAC-seq further validated DMFO’s role in shaping the epigenomic landscape in T_H_17 cells towards a more Treg phenotype. Building on their ATAC-seq data, the authors looked for putative TF binding sites overlapping with chromatin regions whose accessibility was modulated by DFMO. While several TFs were identified, one that stood out was JMJD3, a histone demethylase with known functions in T cell plasticity and modulation of IL-17A expression [18]. CD4^+^ T cell knockout of JMJD3 restricted the Treg program, promoting T_H_17p differentiation. These data are consistent with previous reports that CD4^+^ T cell-specific JMJD3 ablation inhibits Treg differentiation in favor of T_H_17 and T_H_2 phenotypes [19].

Given their data that the polyamine pathway regulates Foxp3 expression during T_H_17 differentiation in vitro, the authors explored how perturbing rate-limiting polyamine enzymes in vivo affected T_H_17-mediated disease development. Experimental autoimmune encephalitis (EAE) is a commonly used mouse model of MS and a T_H_17-driven disease. The authors first demonstrated that mice treated with DMFO displayed reduced severity of disease. Analysis of T cells demonstrated that antigen-specific recall responses were reduced from the draining lymph nodes of DMFO-treated mice and an increase in Foxp3^+^ T cells was observed in the CNS of DMFO treated animals. However, outside of these parameters, no other T cell populations were discussed. The authors also developed CD4^+^ T cell-specific knockouts of the SAT1 enzyme, which displayed delayed onset and decreased EAE disease severity, reduced immune cell infiltration, and increased Foxp3^+^/decreased RORγt^+^ and Tbet^+^ T cells in the CNS at peak of disease. These in vivo data highlight the polyamine pathway as a potential target for autoimmune therapies. However, more work needs to be done exploring whether these prophylactic effects are also seen with ODC1 and SAT1 targeting after disease onset, given that most MS patients do not receive treatment until after substantial disease progression. Administering DMFO or ablating *Sat1*, using an approach that would occur after disease onset, would provide better insight to their therapeutic potential.

Polyamine metabolism is known to play a role in T cell activation [20], T helper cell differentiation [21], and autoimmunity [20]. Compass predicted metabolic regulators, specific enzymatic reactions which also were responsible for regulating the epigenome that ultimately affected the T_H_17/Treg balance. These predictions were validated with chemical and genetic modulation both in vitro and in vivo, demonstrating the impact of polyamine metabolism in the development of EAE (Figure 1). It will be important to determine whether these effects and modulation of this pathway translate to human T_H_17 cells and autoimmunity. Compass has limitations in its predictive power based on available annotated metabolic functions. Additionally, its algorithm does not take into account post-transcriptional and post-translational modifications involved in metabolic regulation. Despite this, Compass correctly predicted the role of aerobic glycolysis in T_H_17p and the role of beta-oxidation in T_H_17n cells. It demonstrated utility by its prediction of novel metabolic processes correlated to the pathogenic severity of T_H_17 cells. Considering the current limitations of unbiased metabolomics research [11], Compass has filled an informatic niche that will guide immunometabolism research for years to come.

## Figures and Tables

**Figure 1.**
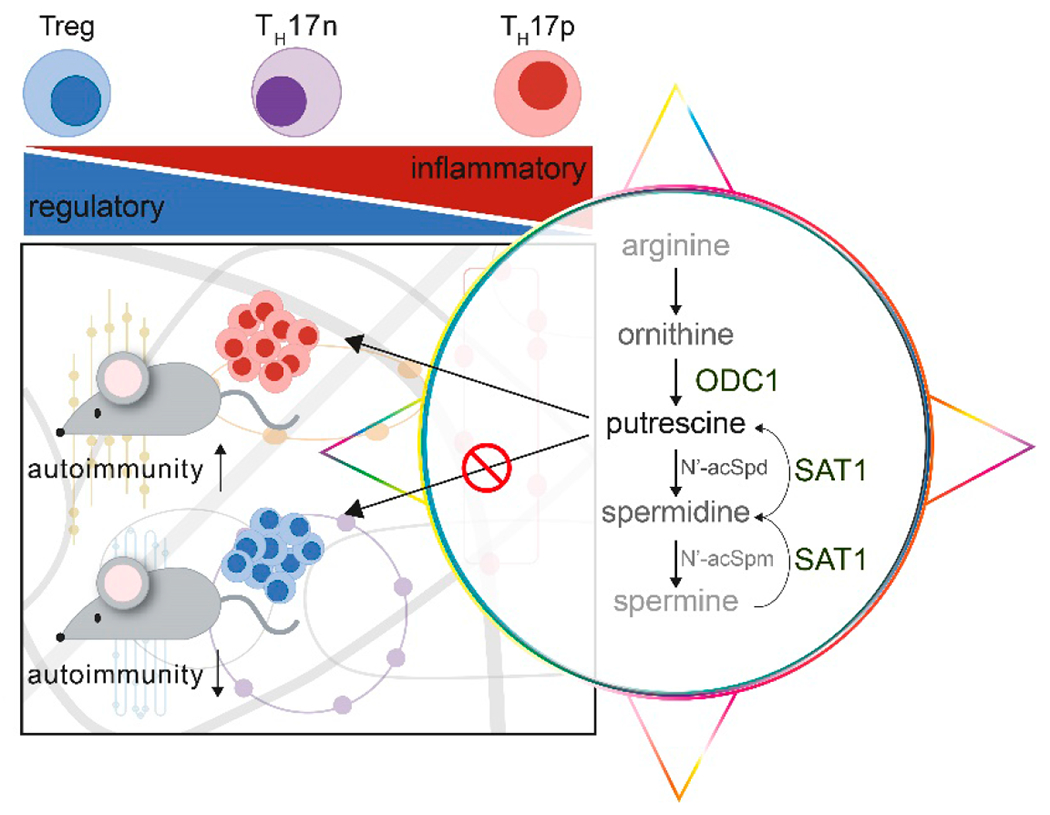
Compass, a flux balance analysis algorithm, was developed to analyze cellular metabolic states at the single cell level. Application of Compass revealed known metabolic switches between T_H_17/Treg cell fates and identified the pathogenic potential of T_H_17 cells through particular metabolic programs. Specifically, Compass revealed the polyamine metabolic pathway was significantly associated with differences in T_H_17 pathogenicity. Alterations in the polyamine pathway affects the development of autoimmunity, favoring a shift in T cell populations towards a more T regulatory phenotype.

## ABBREVIATIONS

T_H_17: T helper 17
MS: multiple sclerosis
EAE: experimental autoimmune encephalitis
Treg: T regulatory cell
RA: Rheumatoid arthritis
scRNA-seq: single cell RNA-sequencing
TF: transcription factor
